# Don’t Ignore the Tipping Point: A Framework to Fuel Diversity, Equity, and Inclusion Efforts in Health-care Sciences

**DOI:** 10.4103/ehp.ehp_11_21

**Published:** 2021

**Authors:** Catherine R. Hoyt, Adam Cisroe Pearson, Jordan Skowronski, Stephanie Lancaster, Cristina Reyes Smith, Andy G. S. Daniel

**Affiliations:** 1 Program in Occupational Therapy, Washington University School of Medicine, St. Louis, MO, USA; 2 Department of Pediatrics, Washington University School of Medicine, St. Louis, MO, USA; 3 Department of Neurology, Washington University School of Medicine, St. Louis, MO, USA; 4 Peter and Paul Community Services, St. Louis, MO, USA; 5 School of Public Health, University of Illinois at Chicago, Chicago, IL, USA; 6 Department of Occupational Therapy, University of Tennessee Health Science Center, Memphis, TN, USA; 7 Division of Occupational Therapy, Medical University of South Carolina, Charleston, SC, USA; 8 Department of Biomedical Engineering, Washington University in St. Louis, St. Louis, MO, USA

**Keywords:** Diversity, equity, inclusion, occupational therapy

## Abstract

In the United States, social determinants of health have resulted in inequities in health care, particularly for communities affected by systemic forms of racism and oppression. Health-care professionals are essential in assisting participation in the health-care system. Building a more diverse health-care workforce can expand access to care, improve the quality of care, and increase health equity. However, many health-care professions have struggled to effectively recruit and retain individuals from diverse backgrounds. In the field of occupational therapy, leaders in diversity, equity, and inclusion worked together to establish a framework with nationwide initiatives to effectively recruit and retain a workforce that is more representative of the communities, in which health-care professionals serve. The initiatives and framework outlined below can be replicated to advance efforts to increase diversity and create systems that address justice, equity, and inclusion.

## Introduction

As the nation continues to grapple with pernicious racial inequities, many have begun to understand that oppression and discrimination of minoritized communities has a negative impact on health and results in disparities in health outcomes.^[1,2]^ Health-care systems and workers operate within these inherently racist structures.^[3]^ While we strive to provide the best care possible, all individuals are influenced by personal experience and subject to unconscious biases that can impact the way that we provide care.^[2]^ Individuals can take steps to understand how their own biases may influence treatment, but it is essential that academic and professional organizations take direct and immediate action to build a health-care workforce that is more representative of the national population.^[4]^ Academic programs share a responsibility with professional associations to promote and advocate for justice, equity, diversity, and inclusion (JEDI) in a manner that is responsive to the needs of the populations they ultimately serve. Rehabilitation professions, such as occupational therapy (OT), provide health-care services to individuals that represent the diversity of the population in the hospital, in private homes, and in the community. In OT, however, only about 17% identify as non-White and only 5% identify as Black or African–American, far below the national population.^[5,6]^ As such, identifying action-oriented strategies to increase diversity in the OT workforce is a primary target for academic programs that aim to increase cultural humility and reduce disparities in access to quality care.^[7]^

In 2014, a grassroots organization, called the Coalition of Occupational Therapy Advocates for Diversity (COTAD, www.cotad.org), was formed to candidly discuss challenges that minoritized students and practitioners faced in OT and develop actionable initiatives to combat these barriers. Here, we share the steps that COTAD used to develop programmatic changes and national visibility in OT. We anticipate that these strategies can be adapted to other health-care fields to continue to build a health-care workforce that is representative of the communities we serve and reflective of the ideals we strive to live by.

## Formation

Mentorship is a well-recognized strategy to build leadership skills and support recruitment and retention efforts, particularly for underrepresented populations.^[8]^ The founding members of COTAD were brought together through an emerging leaders development program hosted by the American Occupational Therapy Association (AOTA) and were encouraged to share their experiences in OT related to their different cultural backgrounds. Since that time, COTAD has grown tremendously, from six founding members to over 4500 followers from countries around the world. While the coalition began to grow, organization and leadership was determined using a consensus process. All members worked on projects where they had personal interest when collectively working toward a common goal. As a volunteer leadership organization, COTAD has thrived by encouraging members to work on projects they are most passionate about. This structure has made it possible to recruit new members for specific purposes and to fill volunteer and leadership needs.

As an established 501c-3 nonprofit organization, COTAD is focused on working toward a common goal of promoting JEDI within OT education, clinical practice, and research. COTAD operates as an independent entity; however, its vision, mission, and platform complement those of our national association, AOTA, which provided opportunities to increase visibility of COTAD during national conferences and meetings. The strong impact of COTAD’s work has shown that for substantive, long-lasting change in health care, the approach to addressing issues around JEDI must be multilevel and the effort must come from both a top-down and a bottom-up approach.

## Development

**Figure 1:**
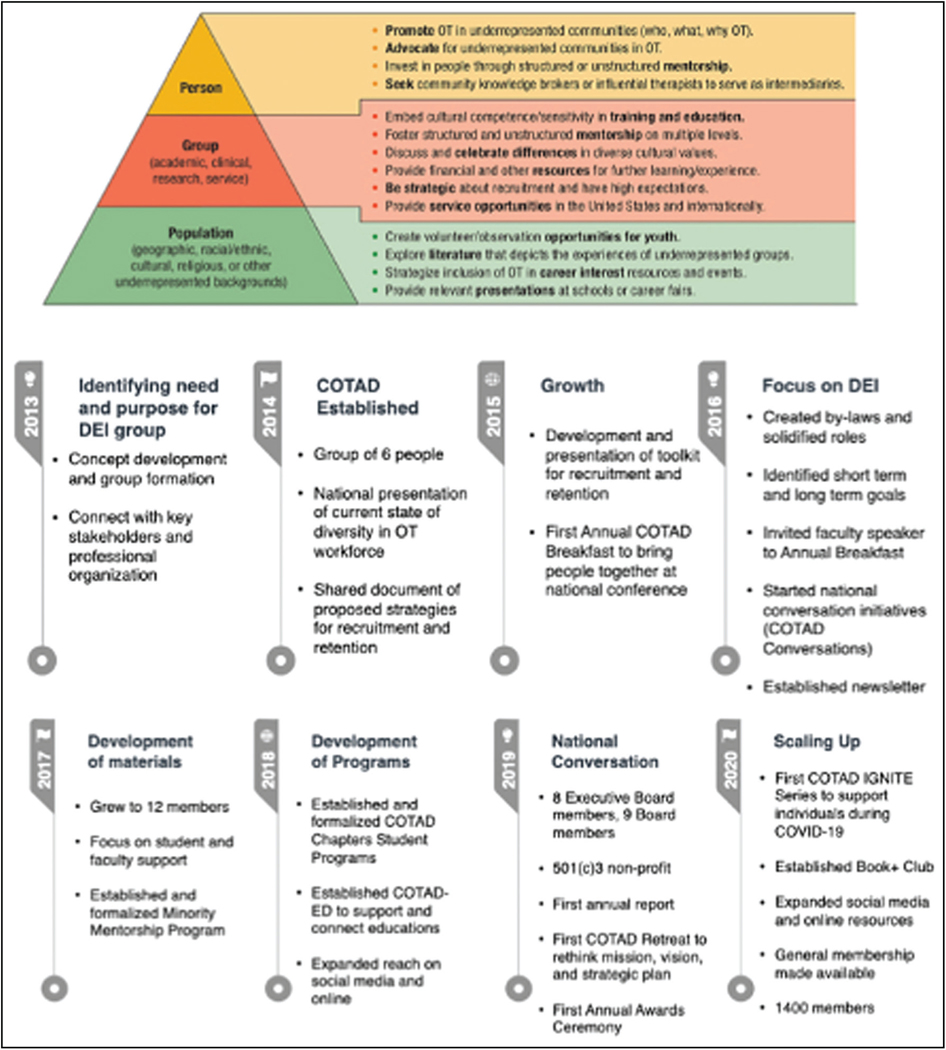
Coalition of Occupational Therapy Advocates for Diversity framework and development timeline^[13]^

One of the first steps COTAD took was identifying key stakeholders that could help amplify the message and connect individuals to help increase visibility in the profession.^[9–12]^ It was critical to bring like-minded people of diverse experiences, willing to dedicate time and lead efforts in JEDI together. COTAD aligned with AOTA but has remained independent to allow for more rapid development and implementation of initiatives. COTAD developed a website and solicited feedback from students, educators, and practitioners about what concerns and ideas they had. Simultaneously, we conducted a nationwide needs assessment at six clinical and academic sites to identify the perceived needs for discussions on JEDI and desired style of support. Through this initial investigation, COTAD identified that the majority of the workforce was interested in learning more about JEDI, but many did not yet have much experience with considering how JEDI might relate to professional development. COTAD learned that many students that identified as part of a minoritized group sought a safe space in their academic environment to openly discuss issues related to diversity and inclusion and early career professionals were eager to engage in one-on-one mentorship with someone who had a similar lived experience. Others communicated a need for opportunities to hear the stories of others and access to materials (e.g., case studies) that exemplified greater diversity. These lessons translated directly to the development of a framework that guides the core directives of COTAD [Figure 1].

The framework grew out of the synthesis of experience with evidence, current education approaches, and the initiatives of AOTA.^[14,15]^

COTAD has used this framework to pilot and establish national programs that address the individual, community, and population to increase representation and inclusion in OT. At the individual level, COTAD has facilitated self-reflection through national presentation with tools such as the implicit bias test. Programs such as the COTAD Mentorship Program are designed to provide individual support to individuals seeking to join OT or who are early career professionals. At the community level, COTAD members have presented about OT at local magnet schools and youth groups. The COTAD Chapters program is an example of a community level initiative where students are supported in creating a local chapter with their faculty to provide a safe space for discussion and opportunities for students to engage their program in discussions to increase representation and inclusion. At the population level, COTAD has advocated for holistic admissions, wording related to a diverse workforce in the profession’s vision statement and strategic plan, and JEDI-related learning objectives to practitioner and fieldwork/internship educator training. Our members hold elected positions in AOTA Board of Directors and Representative Assembly. Beyond the profession, actions include collaborating with other professions to eliminate structural and social determinants of health inequities, which shape education and employment opportunities. As an exemplar, COTAD worked with AOTA to support the Allied Health Workforce Diversity Act of 2019 (H.R. 3637, now H.R. 2781).

## Conclusion

Health-care providers are tasked with identifying solutions to actively combat systemic injustices to improve the quality of health care and quality of life for all. In the seven years that COTAD has existed, it has taken actions at each level and has shown JEDI-rooted improvements in recruitment, retention, education, and within our profession as a whole. Here, we outlined a developing model organization. We recommend that other health-care disciplines make adjustments and modifications to fit their needs. The concepts of JEDI and the use of deliberate and strategic approaches to its implementation from the individual to population level are universal. Using the COTAD model, we propose that individuals in all health-care fields can begin to identify the unique barriers that hinder progress toward providing more equitable care. The current climate toward racial justice behooves a reevaluation of the discipline-specific actions employed to address disparities between members and the communities they serve. It takes the efforts of committed individuals on the ground to catalyze change as well as an environment that is ripe for change. Let us use this momentum to make a tangible impact in our disciplines and the lives of our community members.
